# Supplementary material for: Tasting to preserve: An educational activity to promote children’s positive attitudes towards intraspecific diversity conservation
Source: PLoS One. 2024 Jan 10;19(1):e0285649. doi: 10.1371/journal.pone.0285649 (PMC10781109; doi:10.1371/journal.pone.0285649)
Supplement: S1 Table — Asterisks (*) denote post-test values that significantly differ from pretests according to McNemar test results (p < .05). K1 to K8 are different biology and health knowledge topics addressed in the educational activity. Namely, K1—Different varieties of a vegetal species have different properties (different varieties have distinct tastes, different varieties may have distinct features that make them more suitable for distinct dishes, different varieties have different nutritional properties and make our diet more diverse); K2—Different varieties may grow and produce differently in distinct environments; K3—The food’s degree of ripeness alters its flavor; K4—Different people have distinct tastes and preferences; K5—It is healthy to eat distinct varieties of a vegetable and/or it is not healthy to always eat the same variety of a vegetable; K6—Our tastes change over time, so we should try different varieties of vegetables; K7—The way we chew food influences the taste we get from it; K8—The fact that an individual is sick can change the way they taste food. (DOCX) [file pone.0285649.s001.docx]

**S1 Table.** **McNemar tests results to test for significant differences between pre- and post-tests results for each of the categories of analysis in control and target groups.**

|  | | Target group | | | Control group | | |
| --- | --- | --- | --- | --- | --- | --- | --- |
|  |  | Test statistic | Degree of freedom | Exact sig. | Test statistic | Degree of freedom | Exact sig. |
| Behavioral component | Behavioral intention (biodiverse bag choice) | 6,125 | 1 | **0,008*** | 0,8 | 1 | 0,375 |
| Affective component | Emotional | 2,5 | 1 | 0,109 | 0,25 | 1 | 0,625 |
|  | Aesthetic | 4,9 | 1 | **0,021*** | 0,5 | 1 | 0,5 |
|  | Social/Cultural | 0,8 | 1 | 0,375 | 0 | 1 | 1 |
| Cognitive component | Biology and health knowledge | 6,125 | 1 | **0,008*** | 0,167 | 1 | 0,688 |
|  | K1 | 3,2 | 1 | 0,063 | 0,167 | 1 | 0,688 |
|  | K2 | - | - | - | - | - | - |
|  | K3 | - | - | - | - | - | - |
|  | K4 | 1,333 | 1 | 0,25 | - | - | - |
|  | K5 | 3,2 | 1 | 0,063 | 0 | 1 | 1 |
|  | K6 | - | - | - | - | - | - |
|  | K7 | - | - | - | - | - | - |
|  | K8 | - | - | - | - | - | - |
|  | Economic knowledge | 0,5 | 1 | 0,5 | 3,2 | 1 | 0,063 |
|  | Ethical knowledge | 1,333 | 1 | 0,25 | 0 | 1 | 1 |

Legend: Asterisks (*) denote post-test values that significantly differ from pretests according to McNemar test results (p < .05). K1 to K8 are different biology and health knowledge topics addressed in the educational activity. Namely, K1 - Different varieties of a vegetal species have different properties (different varieties have distinct tastes, different varieties may have distinct features that make them more suitable for distinct dishes, different varieties have different nutritional properties and make our diet more diverse); K2 - Different varieties may grow and produce differently in distinct environments; K3 - The food's degree of ripeness alters its flavor; K4 - Different people have distinct tastes and preferences; K5 - It is healthy to eat distinct varieties of a vegetable and/or it is not healthy to always eat the same variety of a vegetable; K6 - Our tastes change over time, so we should try different varieties of vegetables; K7 - The way we chew food influences the taste we get from it; K8 - The fact that an individual is sick can change the way they taste food.
