# Supplementary material for: Tasting to preserve: An educational activity to promote children’s positive attitudes towards intraspecific diversity conservation
Source: PLoS One. 2024 Jan 10;19(1):e0285649. doi: 10.1371/journal.pone.0285649 (PMC10781109; doi:10.1371/journal.pone.0285649)
Supplement: S3 Table — Underscore (_) denotes that no examples of the specific topic of the ‘biology and health knowledge’ factor were found in the students’ answers. (DOCX) [file pone.0285649.s003.docx]

**S3 Table. Description and examples of the various topics of the 'biology and health knowledge’ factor.**

| Biology and health knowledge | Description | Examples |
| --- | --- | --- |
| The student justifies his/her choice by referring to biology and health knowledge addressed in the educational activity (from topics K1 to K8) | K1 - Different varieties have different properties: i) Different varieties have **distinct tastes**; ii) Different varieties may have distinct features that make them more suitable for **distinct dishes**; iii) Different varieties have **different nutritional properties** and make our diet more diverse. | “because if we always eat the same thing, we don't know what the others taste like”; “when we want one dish we want it with one kind of bean, when we want another dish we want it with another kind of bean”; |
|  | K2 - Different varieties may **grow and produce differently** in distinct environments; | _ |
|  | K3 - The food's **degree of ripeness** alters its flavor; | _ |
|  | K4 - Different **people** have **distinct tastes and preferences**. | “there could be people who would like some and others who would like others”; |
|  | K5 - It is **healthy to eat distinct varieties** of a vegetable and/or it is not healthy to always eat the same variety of a vegetable. | “eating too much of the same thing is bad for you”, “because this way I have a different diet and I'm not always eating the same things”, “because if you eat all kinds it is good for you”; |
|  | K6 - Our tastes change over time, so we should **try different varieties** of vegetables; | _ |
|  | K7 - The **way we chew** food influences the taste we get from it; | _ |
|  | K8 - The fact that an individual is **sick** can change the way they taste food; | _ |

Legend: Underscore (_) denotes that no examples of the specific topic of the ‘biology and health knowledge’ factor were found in the students' answers.
